# Assessing the Effects of Information System Quality and Relationship Quality on Continuance Intention in E-Tourism

**DOI:** 10.3390/ijerph17010174

**Published:** 2019-12-25

**Authors:** Ni Wayan Masri, Jun-Jer You, Athapol Ruangkanjanases, Shih-Chih Chen, Chia-I Pan

**Affiliations:** 1 College of Management, National Kaohsiung University of Science and Technology No. 1, University Rd., Yanchao Dist., Kaohsiung City 824, Taiwan; anandamallika1@gmail.com; 2 Department of Artificial Intelligence CTBC Business School No. 600, Sec. 3, Taijiang Blvd., Annan District, Tainan 709, Taiwan; youjunjer@ctbc.edu.tw; 3 Chulalongkorn Business School, Chulalongkorn University, Bangkok 10330, Thailand; 4 Department of Information Management, National Kaohsiung University of Science and Technology No. 1, University Rd., Yanchao Dist., Kaohsiung City 824, Taiwan; scchen@nkust.edu.tw; 5 Department of Business Management, National Sun Yat-sen University 70 Lienhai Rd., Kaohsiung 80424, Taiwan; d004010008@student.nsysu.edu.tw

**Keywords:** e-tourism, information system quality, perceived value, relationship quality, continuance intention

## Abstract

The advance of electronic commerce has resulted in successful e-travel services. Through the development of e-travel information, consumers can plan their trip without time and space limitations. This study proposes a model regarding the formation of the relationship quality (customer satisfaction and trust), information system quality, perceived value, and customers’ intention to continue in the e-tourism environment. The study is based on 351 e-travel users in Taiwan. The result shows that customer satisfaction has a positive effect on continuance intention. Information system quality has a positive relationship with customer satisfaction, trust, and customer continuance intention. Furthermore, the perceived value has an effect on customer satisfaction and trust. However, the perceived value is partially related to customer continuance intention through customer satisfaction. The managerial implications of this study are discussed.

## 1. Introduction

The success of electronic commerce (EC) is dependent on the internet infrastructure online services [1]. A new model of communication via e-mail, internet, e-travel, web services, and social media has increased in customer service, and the role of traditional communications such as the telephone has decreased [2]. The high-tech environments enable transactions to take place through virtual channels, no longer requiring the physical presence between customers and service providers. The trend is away from face-to-face contact and toward online services [2,3].

A focus on developing online consumers is central to business models in electronic commerce [4]. Travel agents and managers must learn how to maintain customer relationship quality and continuance intention, and they must understand the influence of antecedent factors in the e-tourism environment. Specifically, e-tourism has been rapidly rising in competition around the globe, and therefore many emerging agents have switched from business-to-business (B2B), business-to-consumer (B2C) and Business-to-Business-Consumer (B2BC) in order to sustain their existing customers.

Although the key role of relationship quality related to customer continuance intention has been previously studied, many critical issues still require research, including the formation of relationship quality [5] and customer continuance purchasing behavior to sustain existing customer loyalty in the e-tourism environment. It is clear that some critical factor needs to be developed to enhance customers’ continuance intention in the e-tourism environment. Previous studies confirmed that customer loyalty was found to be directly influenced by customer satisfaction [6,7].

In the present study, we are focused on how the relationship quality (customer satisfaction and trust) is influenced by the information system quality and the customer’s perceived value in the e-tourism environment. The study contributes to the e-tourism literature by extending previous studies and presenting a new concept on relationship quality and information system quality in the e-tourism context. The new construct of relationship quality consists of satisfaction and trust. The three components of information system quality—the information system, system quality, and service quality—are examined as a single construct to enhance the sustainable e-tourism environment. In Section 2, we present the literature review regarding the formation of relationship quality, information system quality, hypotheses, and a summary of our hypotheses in Table 1. The research methodology is in Section 3, our findings are in Section 4, and discussions and implications are in Section 5.

## 2. Theoretical and Hypotheses Development

### 2.1. Relationship Quality

Previous studies have widely investigated relationship quality from different angles [5,8]. Relationship quality is recognized as a key to developing customer loyalty [7,9,10], as well as a number of different constructs related to satisfaction [8] and trust [6,7]. However, different authors have presented combinations of different constructs to indicate relationship quality. Two distinct dimensions of relationship quality (e.g., information sharing and communication quality) have been found to influence long-term customer satisfaction. Further, a recent study found that relationship quality consisted of customer satisfaction, service quality influence, customers’ repurchase intentions, and subjective well-being [11]. The study suggests that relationship quality is a central issue for long-term success in management and business relationships [12]. Relationship quality is the factor that enhances profitability for both parties [13]. Therefore, relationship quality can be posited as an antecedent for customer continuance intentions [11]. Studies have proposed two dimensions of relationship quality (satisfaction and trust) as an antecedent of customer continuance intentions in the e-tourism context. Information system quality and customer perceived value are considered as the antecedents of relationship quality. Moreover, relationship quality affects customer satisfaction and trust, influencing the customer’s continuance to purchase the product or service in the e-tourism environment. We define relationship quality as the customers’ satisfaction and trust relationship toward the information system quality and the perceived product value and service. We define customer satisfaction as the customer perceived value of the information system quality that is provided by an e-tourism provider. We define customer trust as a customer’s subjective belief that an e-travel agency can serve their needs and expectations.

### 2.2. Relationship Quality and Continuance Intention in E-Tourism

According to the expectation disconfirmation model, a customer’s continuance intention is influenced by service quality and customer satisfaction [14]. In an e-commerce B2C model, it has been shown that customer satisfaction influences consumers’ continuance intentions as the outcome of cognitive, affective, and conative loyalty [15]. Furthermore, relationship quality has a positive influence on repurchase intention in B2B e-commerce [16]. Satisfaction and trust have been examined as a single entity, where the relationship quality was considered as a second-order construct [16]. The present study shows that relationship quality consists of satisfaction and trust, which can predict a customer’s continuance to purchase the product or service in the e-tourism environment. Customer satisfaction and trust are examined as separate entities to fit the advanced technology available. For instance, many customers may be satisfied with the information on a website, but this does not mean that the customer will trust the product or service provided. Based on the above literature, our study hypotheses are:

**Hypothesis** **1** **(H1).** Customer satisfaction has a positive effect on continuance intention.

**Hypothesis** **2** **(H2).** Customer trust has a positive effect on continuance intention.

### 2.3. Information System Quality

The information system was proposed in [17]. The study introduced the model, which posits three major dimensions: system quality, information quality, and user satisfaction in the context of organizations. Ten years later, in an update to the original Information System literature, the authors added service quality to the information system model to evaluate the information system through seven factors: information quality, system quality, service quality, information use, use, user satisfaction, and net benefits [18]. This develops a different definition regarding the information system [19]. The system quality consists of response time, system reliability, and system availability, which have a positive impact on the perceived ease of use and usefulness of website [18,20].

Service quality is defined as the user’s perceptions regarding the service performance [21]. Service quality measures the discrepancy between what the customer feels and needs and what is offered accordingly to fulfill the customer’s expectations [20,22]. For example, service quality on multi communication mechanisms enables the user to have their complaints responded to in a timely manner.

**Figure 1:**
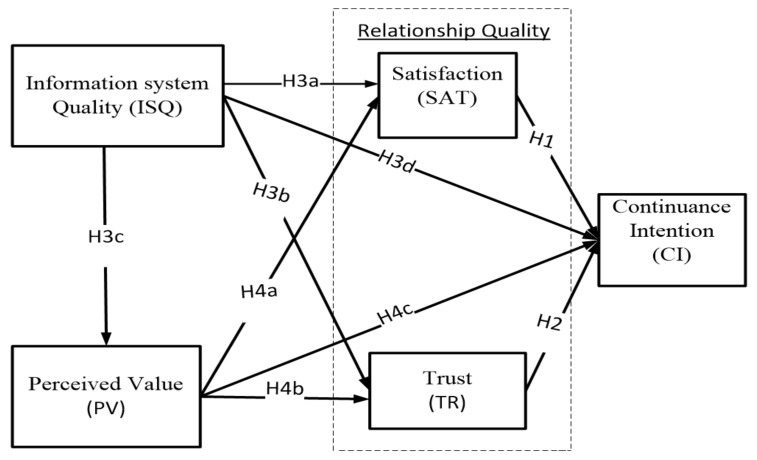
Relationship quality research model. Note: H = hypothesis.

Information system success has been measured in four dimensions: completeness, accuracy, format, and currency [23]. For information quality, the user-perceived effectiveness of system quality measurements have included accuracy, relevance, adequacy, and included quality, timeliness, and sequencing [24]. Other studies have also shown that the information system quality has a significant relationship with perceived usefulness [19]. However, the numbers of previous studies have developed the information system quality in an e-commerce environment. This study adopts the model from the study in [18], as shown in Figure 1. We conceptualize three dimensions of system quality, information quality, and service quality as information system quality to adopt the advanced technology in the e-tourism environment. The information system quality is defined as products or services that fit customer needs and expectations to complete their transaction in the e-tourism environment. The products or services include itinerary services, reliable information, instant information, accurate operation, and specific information with easy access at anytime and anyplace by the customer. For instant, low-cost travel, travel agencies provide interconnected systems, such as TripAdvisor, ezfly.com, or skyscanner.com.tw.

The value that a service involves is not only by the provider but also through the opportunity for customer satisfaction and a trust relationship. The information system quality accommodates the swift customer mindset that has changed from traditional to online 24-h services. The travel agency reduces the time and travel expense, which benefits both parties. Our model has shown that customer trust and satisfaction are influenced by the information system quality and perceived value. The information system quality enhances the mindset of the customer relationship through a single entity of the product and service in an e-travel environment. Perceived value is dependent on the consumer’s perceptions of what is received and what is given [25]. The customer’s perceived value influences the customer satisfaction and trust regarding the product or service in the e-tourism environment. Based on the studies above, we propose the following hypotheses:

**Hypothesis** **3** **(H3).** Information system quality has an effect on customer (a) satisfaction, (b) trust, (c) perceived value, and (d) continuance intention.

### 2.4. Perceived Value

The concept of value has constantly emerged from the different studies related to consumer behavior [25,26]. Prior studies suggested that perceived value is a better predictor of repurchase intentions than satisfaction, commitment, or trust [26]. Furthermore, the perceived value of the product and service could attract new consumers and result in benefits to the vendor [6,27,28]. Our model has shown that customer satisfaction and trust relationships are influenced by the customer’s perceived product and service value. The customer’s perceived value influences the customer’s continuance intention. Based on this literature, we propose the following hypothesis:

**Hypothesis** **4** **(H4).** Perceived value has a positive effect on customer (a) satisfaction, (b) trust, and (c) continuance intention.

## 3. Research Methodology

### 3.1. Subjects: Instrument Development and Measurement

The survey study was designed based on the previous study on the information system success and IT development in an e-commerce environment, looking at service quality, system quality, perceived quality, trust, satisfaction, and continuance of use. We developed and reworded the survey items to fit the present study. All the items in the questionnaire were modified from English, translated to Chinese, and then translated back to English. The initial version of the survey study was pretested by two Ph.D. students and one professor who is an expert in the field of questionnaire design for e-commerce studies. After obtaining feedback from the experts, we modified the questionnaires for the final measurements of the model.

The constructs are measured using five-point Likert scales, ranging from 1–5, with 1 indicating “strongly disagrees” and 5 indicating “strongly agrees”. Information system quality was adopted from [14,18], with seven items. Perceived value, with five items, was modified from [26,28], and three items were taken for further data analysis. Customer trust, with four items, was modified from [6,29]. Satisfaction, with four items, was modified from [6]. Continuance intention was adapted from [30] with five items, and three items were taken for further data analysis, as the loading value was lower than the effective value.

### 3.2. Survey Collection

The questionnaire was targeted to online travel users such as students, information technology users, manufacturers, and customers in finance, public service, and medical fields, who have experience buying domestic or international travel itineraries such as air tickets, reservations, and car rentals, as well as experience booking hotels and other services in e-travel (e.g., Ezfly, Eztravel, Skyscanner, tripadvisor). We chose websites that were the most popular and where the systematic system can easily be accessed by personal computer (PC) and cell phones, with flexible times and affordable prices for young or elderly people. The data were collected over two months in Taiwan. For more information regarding the survey items, refer to Appendix A. To maximize the respondents’ awareness on this survey, we contacted the respondents by email or sent the questionnaire on personal Facebook chat, Line chat, group Facebook, or Line chat. We distributed 450 questionnaires, and 376 were returned, with a response rate of 83.5%. After data sterilization, 25 respondents were dropped due to incomplete responses on the survey. The final sample employed in our study was 351 responses (93.4% of the total responses). We employed IBM SPSS 20 (Armonk, NY, USA) for descriptive analysis to assess the frequency, and the percent range of populations is shown in Table 2.

The majority of respondents used the online travel service itinerary for their travels, which showed that 59.3% were female and 40.7% were male. The participants were mostly from the age groups of 21–30 years old and 51–60 years old. The participants mostly work in manufacturing and public service from different sectors. The highest monthly income was 1001–2000 USD. The participants searched for information using a smartphone 67.5% of the time. Further, eztravel (29.3%) and TripAdvisor (24.2%) were the most popular e-travel service websites for finding information and booking itineraries. The data samples were sufficient to identify the customers’ behavior and to accommodate further study on the information system quality.

## 4. Data Analysis Results

### 4.1. Measurement Model Analysis

The measurement model and structural model was assessed with partial least squares (PLS) using Smarts-PLS 3.2.8 [31]. First, PLS is not as restrictive on the sample size as that designed in the structural equation model. The constructs in this study are all reflective. Therefore, a PLS modeling approach was chosen in this study.

Second, for the data analysis, we started with the PLS algorithm that can obtain at convergence, satisfying fixed-point equations which include measurement reliability and model validity.

The third, the bootstrap procedure [32] was used to test the significance of various results such as path coefficients, Cronbach’s alpha, and *R*^2^ values. As in bootstrapping, subsamples are randomly drawn observations from the original set of data. This process was repeated until a large number of random subsamples were created, which in the case of our study was 2000 subsamples. The estimations from the bootstrap subsamples were used to derive standard errors for the PLS-SEM results. With this information, *t*-values, *p*-values, and confidence intervals were calculated to assess the significance of model studies.

Four, confirmatory factor analysis was conducted to assess the item loadings, discriminant validity, and internal consistency of the model. Item loading and internal consistencies greater than 0.70 were considered acceptable [33,34]. Moreover, to assess convergent and discriminant validity, first, the indicators loaded should be stronger than corresponding ones on the other constructs. Second, the square root of the average variance extracted (AVE) should be greater than the internal-constructs correlations shown in Appendix B, (cross-factor loading) which confirms the presence of a valid discriminant. Furthermore, in Table 3, the Cronbach’s alpha values range from 0.91 to 0.98, and the AVE ranges from 0.79 to 0.96, indicating acceptability [33]. These results demonstrate that all the measurements have an adequate acceptability level.

Five, an exploratory factor analysis was conducted to determine the relationship factors in the Smart-PLS algorithms. The exploratory factor analysis results are shown in Table 4. Standard factor loading and the *t*-value on the measurements were significant at the level of 0.01–0.02. Table 5 shows all the items of latent variables correlations on their intended factors to determine if the survey study is adequate for further analysis.

### 4.2. The Results of Structural Model

**Figure 2:**
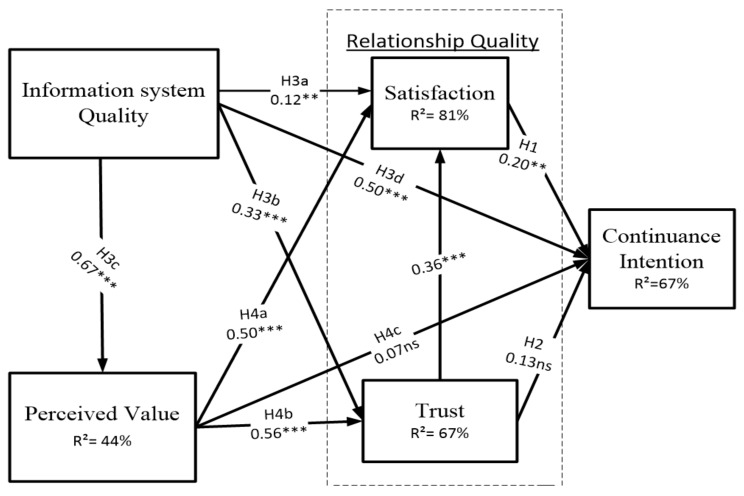
The results of the relationship quality model. Note: ** *p* < 0.01 = *t* > 2.58; *** *p* < 0.001 = *t* > 3.29; with a two-tailed test. ns = not supported.

The results of the Smart-PLS part coefficients and significance values are shown in Figure 2. Table 6 shows the summary of our hypotheses testing. Seven of the nine hypotheses have positive and significant relationships. Customer satisfaction has a positive and significant effect on continuance intention, which supports H1, SAT–CI (β = 0.20, *t* = 2.59, ** *p* < 0.01). However, the influence of customer trust has no significant relationship with continuance intention. Thus, H2 is not supported, trust–continuance intention (TR–CI) (β = 0.13, *t* = 1.89, *p* < 0.05). The testing (H3a, H3b, H3c, and H3d) showed that information system quality has a positive and significant effect on customer satisfaction, trust, perceived value, and customer continuance intention. Thus, H3a, information system quality–satisfaction (ISQ–SAT) (β = 0.12, *t* = 2.99, *** *p* < 0.001), H3b, ISQ–TR (β = 0.33, *t* = 5.82 *** *p* < 0.001), H3c, ISQ–perceived value (PV) (β = 0.67, *t* = 17.0 *** *p* < 0.001), and H3d, ISQ–CI (β = 0.50, *t* = 7.50, *** *p* < 0.001) were supported. Consequently, the (H3a, H4b), customer perceived value has a positive and significant effect on customer satisfaction and customer trust. Thus, H4a and H4b were supported (PV–SAT, β = 0.50, *t* = 8.30, *** *p* < 0.001, and PV–TR, β = 0.56, *t* = 10.1, *** *p* < 0.001). However, perceived value does not have a significant influence on customer continuance intention, and so H4c, PV–CI (β = 0.07, *t* = 0.97, *p* < 0.05) is not supported. The model also explains 67% of the variance of customer continuance intention, 81% of the variance of customer satisfaction, 67% of the variance of a customer trust relationship, and 44% of the variance of customer perceived value of the product or service on an e-tourism channel.

### 4.3. Mediation Effects

To solve the problem in the hypotheses (H2, H4c), this study performed mediating effects following certain steps [35,36]. First, the study tested the significant indirect effect of the product paths “a” and “b”) using the Sobel test [37].

The results showed that perceived value has a positive and significant effect on continuance intention through the mediator of customer satisfaction, PV–SAT–CI, with a Sobel-test statistic (z = 3.10, ** *p* < 0.01). Consequently, customer trust has a positive and significant effect on customer continuance intention through customer satisfaction, TR–SAT–CI, with a Sobel-test statistic (z = 2.31, * *p* < 0.05). Second, the study also accesses the variance-accounted-for (VAF) ratio by accounting effect (indirect effects/total effects = VAF). Thereby, we can determine the extent to which the dependent variable is directly explained by the independent variable and how much of the target construct variance is explained by the indirect relationship via the mediator variable [38,39]. If the VAF ratio is less than 20%, it shows a non-significant mediating effect; when the ratio is 20%–80%, it shows partial mediating effects, and when it is larger than 80%, it is assumed to have a fully mediating effect.

The test results showed that customer perceived value has a partially mediating relationship on continuance intention through the mediator customer satisfaction (PV–SAT–CI), with a variance-accounted-for (VAF) ratio of 75%. Furthermore, customer trust has a partially mediating relationship on customer continuance intention through the mediator of customer satisfaction (TR–SAT–CI) with a variance-accounted-for (VAF) ratio of 35%. The summary of mediating effects is shown in Table 7.

## 5. Discussion

### 5.1. Theoretical Implication

Several implications are obtained from this study. First, this study extends the previously study [18], and the findings support the research on information system quality by examining information quality, system quality, and service quality as a single entity.

Second, the prior study on customer relationship quality examined this quality as a single entity and showed a positive and significant effect on continuance intention [7]. In this study, relationship quality was examined as two separate entities (customer satisfaction and customer trust). Customer satisfaction had a positive and significant effect on the continued use of the products or service in the e-tourism environment. Customers showed a continuance of trust in the products or service when the customer was satisfied with the product or service. The information system quality proved long-term usage investment on customer continuance intention, which differs from the existing studies [18] and supports [30,40] customer relationship quality in the e-tourism environment.

Third, the customer-perceived value has a positive effect on customer satisfaction and trust. However, perceived value has no significant relationship on customer continuous intention. Furthermore, perceived value has a partial influence on customer continuance intention through customer satisfaction. This means that without sufficient customer satisfaction, customers may not tend to purchase in the future or will be unable to retain long-term success in e-tourism. The travel agencies have to build existing customers through customer relationship quality, in particular, building customer satisfaction and trusting relationships.

The findings stated that the customer’s relationship qualities (e.g., trust and satisfaction) are the main issues affecting the continuing usage intention in regard to information system quality. Providing a new model, such as a one-desk information service and improved relationship quality (customer satisfaction and trust) could enhance the impact of information system quality on the e-tourism environment.

Fourth, the study found that the majority of respondents who employ the online travel service for their travels were 59.3% female and 40.7% male. The mean gender show a similar benefit from the information system quality in e-tourism. Moreover, results also showed that the participants from ages 21–30 and 51–60 years old who worked in manufacturing and the public service from different sectors were more expected to continue to purchase the products or service.

Furthermore, the customers with income from 1001–2000 USD more frequently used the information and booking and were the most familiar with the websites Eztravel 29.3% and TripAdvisor 24.2%. However, most customers used a smartphone as their preferred tool for booking travel plans. We conclude that these characteristic customer behaviors are shown to provide continuance success in the information system applications in this e-commerce environment, which is different from the findings of previous studies [18].

### 5.2. Practical Implication

The practical implications for information system quality, perceived value, relationship quality, and customer’s continuance intention offer important implications for travel agencies and managers in e-tourism. To improve IS quality in the e-tourism environment, the travel agency and manager has to upgrade the operational process infrastructure and delivery service transaction to match real-time customer expectations. Furthermore, they require software and hardware with an advanced information system that can prevent technical difficulties and transactions overloading [30].

Managers and practitioners can use these results as guidelines to develop websites, operations, and provide advance support to customers. The measurement of information system quality can enhance products and services to help managers and organizations provide better products and services in the e-tourism environment. In addition, these results may apply a particularly powerful benchmark against competitors’ websites that can affect long-term development activities on e-tourism. Our findings not only support a viewpoint on information system quality development, but also on building customer relationship quality through the application of customer satisfaction and trust provided by the e-tourism provider. Service providers can provide an incentive program such as purchasing a bundle program for customer travel planning. Furthermore, the study also demonstrated the positive and significant influence of perceived value on customers’ continuance intention through customer satisfaction, which enhances long-term customer success in adopting a new program such as a package information service. This may suggest that enhancing information system quality is not only to satisfying for the customers but also helps to build customer trust relationships in an e-tourism environment.

## 6. Conclusions and Future Study

This study has important implications for the researcher and practitioner. The study concludes that customer relationship quality (satisfaction) has positive effects on customer continuance intentions. However, customer trust also has a partial relationship on continuance intention through customer satisfaction. In addition, information system quality has a significant relationship with customer satisfaction, trust, perceived value, and continuance intention. Furthermore, the customer-perceived value is also significantly related to customer satisfaction and trust, but it is partially related to customer continuance intention through the customer satisfaction relationship.

However, nowadays, e-tourism companies have greatly invested in training programs and advertising campaigns to transform information system quality for the users. This study provided more comprehensive findings on the information system and examined three dimensions (information quality, system quality, and service quality) in a single entity as information system quality, but it also separately examined the relationship quality that consists of customer satisfaction and trust in the e-tourism environment. We attempted to integrate perceived value and customer relationship quality with the model on customer continuance intention. Some interesting findings that were not discussed in previous studies are also covered in the current study. A large sample in this study is from the manufacturing and services sector, which is due to the customer trust and satisfaction in e-tourism. This study also provides meaningful implications on e-tourism continuance intention behavior.

A limitation in this study is using self-report instruments, as this may have the potential for a common method bias in measuring the study variables [41]. Hence, we diminished the probability of common biases by segregating the instruments and motivating the participants in the study. Furthermore, study data were collected in Taiwan. The travel forum members have similar culture and convenience traits. More research across countries and cultures will be required in order to generalize the findings. Finally, future studies also have the possibility to investigate different factors that can be integrated into the model.

## Figures and Tables

**Table 1:** Relationship hypotheses.

Hypotheses		Part
H1	SAT has a positive relationship on customer CI	SAT->CI
H2	TR has a positive relationship on customer CI	TR->CI
H3a	ISQ has a positive relationship on customer SAT	ISQ->SAT
H3b	ISQ has a positive relationship on customer TR	ISQ->TR
H3c	ISQ has a positive relationship on customer PV	ISQ->PV
H3d	ISQ has a positive relationship on customer CI	ISQ->CI
H4a	PV has a positive relationship on customer SAT	PV->SAT
H4b	PV has a positive relationship on customer TR	PV->TR
H4c	PV has a positive relationship on customer CI	PV->CI

Note: SAT = satisfaction; CI = continuance intention; TR = trust; ISQ = information system quality; PV = perceived value.

**Table 2:** Demographics of the respondents.

Demographic Respondents (*N* = 351)					
Characteristics	Frequency	Percent (%)	Characteristics	Frequency	Percent (%)
Gender			Occupation		
Male Female	143 208	40.7 59.3	Student Technology Manufacturing Finance Service Medical	52 38 75 56 93 37	14.8 10.8 21.4 16.0 26.5 10.5
Age			Monthly Income		
≤20 21–30 31–40 41–50 51–60 >60	29 89 56 66 78 33	8.3 25.4 16.0 18.8 22.2 9.4	≥1000$ 1001–2000$ 2001–3000$ 3001–4000$ <4000	45 169 49 6 82	12.8 48.1 14.0 1.7 23.4
Education			Travel service		
≥Senior high school University/college Graduate above	70 155 126	19.9 44.2 35.9	hkexpress ezfly eztravel TripAdvisor Others	70 70 103 85 23	19.9 19.9 29.3 24.2 6.6
Instruments					
PC Smartphone	114 237	32.5 67.5			

Note: PC = personal computer.

**Table 3:** Construct reliability and discriminant validity.

Constructs	Items	Cronbach’s Alpha	Composite Reliability	AVE	CI	ISQ	PV	SAT	TR
Continuance Intention	CI	0.98	0.99	0.96	0.98				
Information system Quality	ISQ	0.97	0.98	0.87	0.78	0.93			
Perceived Value	PV	0.95	0.97	0.91	0.67	0.66	0.96		
Satisfaction	SAT	0.91	0.94	0.79	0.72	0.70	0.86	0.89	
Trust	TR	0.92	0.94	0.80	0.70	0.70	0.78	0.83	0.90

Note: AVE = average variance extracted.

**Table 4:** Weight and loading.

Constructs	Items	Outer Loading	Outer Weights	Standard Deviation	T Statistics
Continuance Intention	CI1	0.98	0.34	0.01	171.44
	CI2	0.98	0.34	0.01	161.33
	CI3	0.98	0.33	0.01	137.83
Information system Quality	ISQ1	0.95	0.16	0.01	97.28
	ISQ2	0.94	0.16	0.02	62.09
	ISQ3	0.92	0.15	0.02	49.69
	ISQ4	0.92	0.15	0.01	67.88
	ISQ5	0.93	0.15	0.02	61.26
	ISQ6	0.94	0.15	0.01	101.48
	ISQ7	0.90	0.14	0.02	54.40
Perceived Value	PV3	0.95	0.34	0.01	102.10
	PV4	0.96	0.35	0.01	134.48
	PV5	0.96	0.35	0.01	93.05
Satisfaction	SAT1	0.89	0.31	0.01	63.35
	SAT2	0.90	0.27	0.02	59.68
	SAT3	0.85	0.24	0.02	38.83
	SAT4	0.91	0.31	0.01	62.48
Trust	TR1	0.87	0.31	0.02	57.57
	TR2	0.88	0.27	0.02	52.50
	TR3	0.91	0.26	0.02	55.83
	TR4	0.92	0.28	0.01	69.56

Note: Both the standard deviation and *t*-value are for loading not for weighting.

**Table 5:** Latent variable correlations.

Constructs	Items	CI	ISQ	PV	SAT	TR
Continuance Intention	CI	1.00	0.78	0.67	0.72	0.70
Information System Quality	ISQ	0.78	1.00	0.66	0.70	0.70
Perceived Value	PV	0.67	0.66	1.00	0.86	0.78
Satisfaction	SAT	0.72	0.70	0.86	1.00	0.83
Trust	TR	0.70	0.70	0.78	0.83	1.00

**Table 6:** Summary the results of the hypotheses.

Hypotheses		Path Coefficients	*t*-Value	Results
H1	SAT has a positive effect on customer CI	0.20	2.59	Supported
H2	TR has a positive effect on customer CI	0.13ns	1.88	Not supported
H3a	ISQ has a positive effect on customer SAT	0.12	2.99	Supported
H3b	ISQ has a positive effect on customer TR	0.33	5.82	Supported
H3c	ISQ has a positive effect on customer PV	0.67	17.0	Supported
H3d	ISQ has a positive effect on customer CI	0.50	7.50	Supported
H4a	PV has a positive effect on customer SAT	0.50	8.30	Supported
H4b	PV has a positive effect on customer TR	0.56	10.1	Supported
H4c	PV has a positive effect on customer CI	0.07ns	0.97	Not supported

Note: SAT = satisfaction; CI = continuance intention; TR = trust; ISQ = information system quality; PV = perceived value. ns = not supported.

**Table 7:** Results of mediating effects.

Indirect Effect	IV-MD	MD-DV	C	*c’*	AB	Total Effect	Sobel	VAF%	Type
PV-SAT-CI	0.50 ***	0.20 **	0.20 ***	0.67 ***	0.21 ***	0.28 ***	3.10 **	75%	Partial
TR-SAT-CI	0.36 ***	0.20 **	0.28 ***	0.70 ***	0.07 **	0.20 ***	2.31 *	35%	Partial

Note: IV = independent variable; MD = mediator; DV = dependent variable; VAF = variance-accounted-for; IV-MD = The IV significantly affects the mediator; MD-DV = the mediator has a significant unique effect on the DV; C = the effect of the IV on the DV shrinks upon the addition of the mediator to the model; *c’* = The IV significantly affects the DV in the absence of the mediator; AB = the total indirect effect. * *p* < 0.05 = *t* > 1.96; ** *p* < 0.01 = *t* > 2.58; *** *p* < 0.001 = *t* > 3.29; with one-tailed test.

## Appendix A

**Table A1:** Survey item.

Part 1: Demographic					
*Gender*	*1□ Male 2 □ Female*				
*Age*	*1□≥ 20 2□ 21–30 3□31–40 4□ 41–50 5□ 51–60 6□ < 60*				
*Education*	*1□ ≥Senior high school 2 □ University/college 3 □ Graduate above*				
*Occupation*	*1.□ Student 2□ Technology 3□ Manufacturing 4□ Finance 5□ Service 6 □ Medical*				
*Monthly Income*	*1□≥1000$ 2□1001-2000$ 3□2001–3000$ 4□3001-4000$ 5□ < 4000*				
*Travel service*	*1□ hkexpress 2□ ezfly 3□ eztravel 4□ sky scanner 5□ TripAdvisor 6□ Others*				
*Instrument*	*1□ PC 2 □ Smart phone*				
	Strongly disagree	Disagree	Neutral	Disagree	Strongly agree
Part 2(ISQ): This section to know; How do the information system quality and the website system provided service the on customer online travel.					
Information System quality [14,18,42]					
1. Online travel provider provided the customer with a complete information itinerary	□	□	□	□	□
2. Online travel provider provided travelers a complete reliable information	□	□	□	□	□
3. Online travel provider provided travelers with instantaneous information	□	□	□	□	□
4. Online travel provider provided travelers accurately operators	□	□	□	□	□
5. Online travel provider provided the information what I need	□	□	□	□	□
6. Online travel provider provided the customer with information on specific websites	□	□	□	□	□
7. Online travel provider provided appropriate information	□	□	□	□	□
Part 3(PV): This section to know; How do you feel about the value of service and product provided by online travel agency [26,28,43]					
1. Online travel agency can save more time and travel expenses.	□	□	□	□	□
2. Online travel agency allows me quickly complete of my travel itinerary	□	□	□	□	□
3. Online travel agency, it is helpful to me.	□	□	□	□	□
4. Although it takes some time to compare travel itineraries on the Internet, it is worthwhile to do so.	□	□	□	□	□
5. In short, online travel agency provided more benefit and fast processing the information.	□	□	□	□	□
Part 4: This section to know; 1. How do you trust the service and the products provided by travel agency [6,25,29].					
1. I think the company that provides online travel website and information is reliable.	□	□	□	□	□
2. I think the services provided by online travel operators are trustworthy.	□	□	□	□	□
3. Online travel operators should able to perform services and promised to users	□	□	□	□	□
4. I believe that online travel provider has the ability to protect user	□	□	□	□	□
2. How do you satisfies with the online service provided by travel agency [6,44]					
1. I am satisfied with the travel planning provided by the online travel agency.	□	□	□	□	□
2. I am satisfied with the information provided online on website.	□	□	□	□	□
3. I am sure that the online travel website is such a convenience service	□	□	□	□	□
4. The product or service provided by online travel agency are generally quite profitable	□	□	□	□	□
Part 5 (CI) [9,40]: This section, To know about how your future planning on e-tourism					
1. The overall experience of using online travel websites is enjoyable.	□	□	□	□	□
2. I am willing to use the services provided by online travel agency	□	□	□	□	□
3. I will use the travel website to plan my travel itinerary in the future.	□	□	□	□	□
4. I would like to introduce the travel itinerary to my friends	□	□	□	□	□
5. I am willing to continue to purchase product or service itinerary provided by the online travel provider.	□	□	□	□	□

## Appendix B

**Table A2:** The Result of cross loading.

	Cross Loadings					
Constructs	Items	CI	ISQ	PV	SAT	TR
Continuance Intention	CI1	0.98	0.76	0.68	0.71	0.70
	CI2	0.98	0.77	0.66	0.71	0.69
	CI3	0.98	0.75	0.64	0.69	0.67
Information system Quality	ISQ1	0.76	0.95	0.66	0.68	0.67
	ISQ2	0.76	0.94	0.66	0.69	0.69
	ISQ3	0.72	0.92	0.59	0.63	0.62
	ISQ4	0.70	0.92	0.61	0.64	0.65
	ISQ5	0.71	0.93	0.62	0.66	0.66
	ISQ6	0.73	0.94	0.61	0.66	0.66
	ISQ7	0.68	0.90	0.58	0.61	0.60
Perceived Value	PV3	0.60	0.63	0.95	0.80	0.76
	PV4	0.66	0.64	0.96	0.82	0.75
	PV5	0.66	0.64	0.96	0.84	0.72
Satisfaction	SAT1	0.70	0.66	0.87	0.89	0.73
	SAT2	0.58	0.60	0.72	0.90	0.72
	SAT3	0.51	0.53	0.63	0.85	0.70
	SAT4	0.71	0.68	0.80	0.91	0.78
Trust	TR1	0.69	0.71	0.76	0.83	0.87
	TR2	0.59	0.59	0.66	0.73	0.88
	TR3	0.58	0.55	0.66	0.68	0.91
	TR4	0.63	0.63	0.69	0.71	0.92
